# Supplementary figures and images for: Integrating Taxonomies Into Theory-Based Digital Health Interventions for Behavior Change: A Holistic Framework
Source: JMIR Res Protoc. 2019 Jan 15;8(1):e8055. doi: 10.2196/resprot.8055 (PMC6350087; doi:10.2196/resprot.8055)

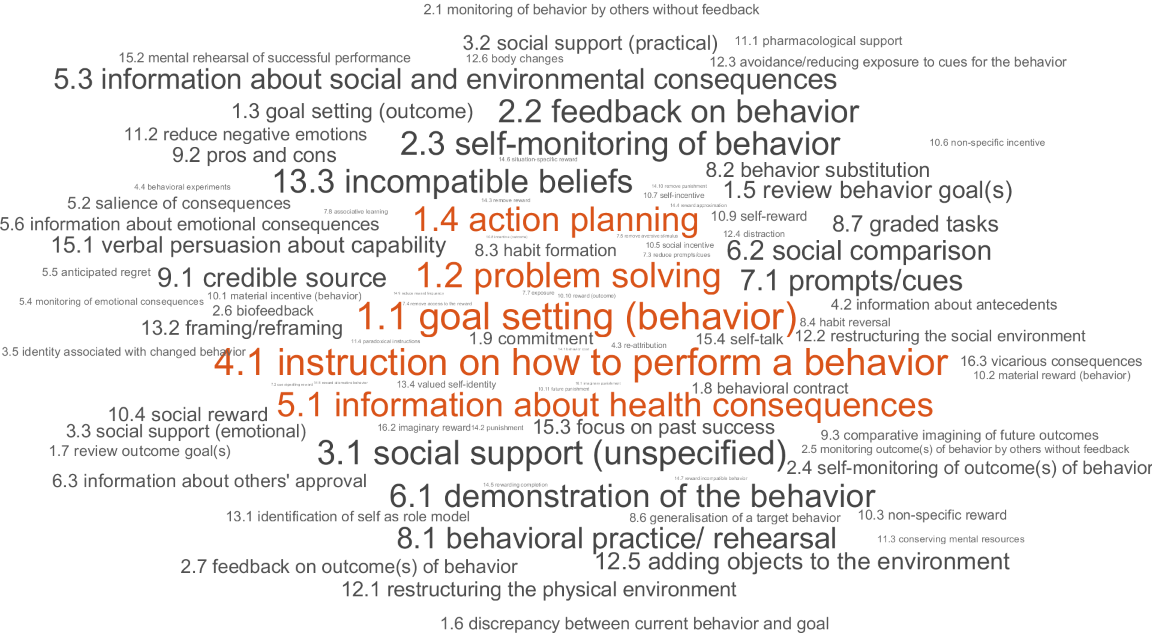

Supplement: Multimedia Appendix 1 [file resprot_v8i1e8055_app1.png]
